# Post-COVID Condition Does Not Alter Cognitive Functions in Young Adults: A Cross-Sectional Study in North India

**DOI:** 10.7759/cureus.82208

**Published:** 2025-04-13

**Authors:** Vatsal A Batra, Kairavi B Unarkat, Manpreet Kaur, Himani Ahluwalia, Soumen Manna

**Affiliations:** 1 Physiology, Vardhman Mahavir Medical College and Safdarjung Hospital, New Delhi, IND

**Keywords:** attention span, executive function, long covid-19, neurocognition, neurology and cognition, neurophysiology, post-covid-19 conditions, visuospatial working memory

## Abstract

Background

Long COVID, or post-COVID condition, includes multi-system chronic sequelae that can last weeks, months, or even years in some individuals after recovery from COVID-19 infection. Prominent among these long-term sequelae are cognitive deficits that may prove to be problematic, especially for the working young adult population. The present study aimed to determine whether cognitive deficits are observed long after recovery from mild COVID-19 infection.

Methods

In this cross-sectional observational study, 29 young adult undergraduate medical students with a history of mild COVID-19 infection at least two years prior were included as cases, while 29 age- and sex-matched undergraduate medical students with no history of COVID-19 were recruited as controls. Sociodemographic data were collected, and the participants were then administered a series of cognitive tests using the National Institutes of Health (NIH) Toolbox V3 software (Toolbox Assessments Inc., Chicago, USA; https://nihtoolbox.org/) to evaluate the cognitive functions, including executive function, cognitive flexibility, attention, working and episodic memory, and processing speed.

Results

The mean age of the cases and controls was 19.37 ± 0.92 and 19.65 ± 0.99 years, respectively. However,there was no statistically significant difference in cognitive function performance across any of the tested domains between cases and controls.

Conclusion

The results of our study indicate that, compared to healthy controls, cognitive functions were not impaired in young adults who previously had symptomatic mild COVID-19 infection.

## Introduction

The cumulative number of cases of COVID-19 as of August 2024 stood at a figure of 776 million, with 45 million of these cases being reported from India [1]. SARS-CoV-2, by virtue of the large number of receptors it can bind to, can enter a variety of cell types [2], resulting in multi-system manifestations involving the respiratory, cardiac, immune, gastrointestinal, nervous, as well as other systems of the body [3]. In light of these varied sequelae of COVID-19 infection, the long COVID syndrome has emerged as a persistent problem in many survivors of SARS-CoV-2 infection. This syndrome is commonly characterized by persistent fatigue, malaise, and dyspnea, as well as joint pain, palpitation, and myalgia, along with many other multi-system manifestations [4].

One of the most concerning effects of this syndrome is the impairment of neurological functions, widely described in the literature [5-7]. The common symptoms of neurological impairment in patients of this syndrome include depressed mood, insomnia, anxiety, memory impairment, attention span deficits, and decreased reaction time [5,8]. Deficits in memory, attention span, and reaction time are particularly concerning issues for young adult working professionals, even more so in the field of medicine. Subjective memory concerns have been shown to reflect objective problems and observable changes in everyday function, affecting daily functioning and resulting in long-haul sequelae of COVID-19 infection [8]. Studies have demonstrated links of both severe as well as mild COVID-19 infection to neurological sequelae; however, these studies were conducted within a period of a few months (<6 months) of recovery from COVID-19 infection [5,9,10]. Nevertheless, there is still a paucity of literature regarding the long-term effects of COVID-19 infection on cognitive function, particularly in young adults with mild COVID-19 infection.

In this study, we assessed the cognitive functions of young adult subjects with a history of mild (not requiring hospitalization) COVID-19 infection and assessed how their cognitive functions compared to those of age- and sex-matched controls. We hypothesized that there would be no significant long-term cognitive differences between recovered COVID-19 patients and controls.

## Materials and methods

Study design

This cross-sectional observational study was carried out over a period of three months (May 2024 to July 2024) at Vardhman Mahavir Medical College and Safdarjung Hospital, a tertiary care facility in New Delhi, India. The study population was selected using convenient sampling based on a survey of young adult undergraduate medical students at the institute. All work on the study was started after obtaining informed consent from the selected participants and clearance from the Institutional Ethics Committee (IEC).

Participants

A survey across batches of the institute was conducted via a comprehensive Google Form (Google LLC, Mountain View, USA) to assess and recruit willing candidates for the study. The results of the survey were used to recruit participants with a history of mild COVID-19 infection (not requiring hospitalization) as well as age- and sex-matched control participants without a prior history of COVID-19 infection. All cases of mild COVID-19 infection considered were based on a positive reverse transcription polymerase chain reaction (RT-PCR) or rapid antigen test results.

Young adult undergraduate medical students aged 18-25 years (male and female) of the institute who had been diagnosed with COVID-19 infection at least two years prior were taken as cases. Age- and sex-matched undergraduate medical students of the same institute who had no history of COVID-19 infection in the past were taken as controls. Any participant with known major medical, surgical, or neurological disease, as well as patients with asymptomatic COVID-19, were excluded from the sample population.

Sample size

The sample size was calculated using a reference from the article by Zhou et al. [7]; the minimum sample size with a confidence interval (2-sided) of 95% and 80% power was 58 (29 in each group). Calculation of sample size was done using G*Power 3.1 software (Heinrich-Heine-Universität Düsseldorf, Düsseldorf, Germany; http://www.gpower.hhu.de).

Procedures

The cases were surveyed for age, socioeconomic status, educational status, smoking history, duration since positive COVID-19 diagnosis, vaccination status at the time of diagnosis and current vaccination status, coexisting disease, treatment received during the course of COVID-19 infection, as well as any lasting symptoms at the time of cognitive testing. The controls were surveyed for the same fields as applicable.

Both cases and controls were subsequently examined using National Institutes of Health (NIH) Toolbox V3 cognition testing software (Toolbox Assessments Inc., Chicago, USA; https://nihtoolbox.org), which is a reliable tool to detect even mild cognitive impairment [11]. This software contains a number of tests across cognitive, motor, sensory, and emotional domains, which can be administered to participants in the age group of 3-85+ years. Two iPads (9th Generation with OS 17.4 and 7th Generation with OS 17.5.1) (Apple Inc., Cupertino, USA) were used for testing. The cognition testing of all participants was carried out in a lab setting at their convenience after ensuring they had taken adequate rest.

Using NIH Toolbox V3 software, the following cognitive battery of tests were administered to each participant: (i) Dimensional Change Card Sort (DCCS) Test: It is an assessment of executive function, cognitive flexibility, and attention span, wherein the participant is asked to match a series of picture pairs to a target picture. (ii) Flanker Inhibitory Control and Attention (FICA) Test: This test is for inhibitory control and attention. The participant is asked to focus on a particular stimulus while inhibiting attention to the stimulus flanking it. (iii) List Sorting Working Memory (LSWM) Test: It is an assessment of working memory. The participant is asked to recall and sequence different stimuli that are presented visually and via audio. (iv) Pattern Comparison Processing Speed (PCPS) Test: It is an assessment of processing speed. Participants are asked to quickly determine whether two stimuli are the same or not the same. (v) Picture Sequence Memory (PSM) Test: It is an assessment of episodic memory. Participants are shown a number of activities, and then asked to reproduce the sequence of pictures in the order it was presented to them. (vi) Fluid Composite Score (FCS): This is a composite score derived by averaging the standard scores and then deriving standard scores based on this new distribution. FCS demonstrates the capacity for new learning, solving problems, and information processing in novel situations. Thus, it is helpful for adapting to novel situations in everyday life. This score has been shown to have an excellent test-retest correlation with r=0.95 [11].

The results of the individual tests in the cognitive battery, as well as the fluid composite score, were obtained in terms of raw score, computed score, change sensitive score (CSS), age-adjusted standard score, and national age-adjusted percentile. 

The age-adjusted standard score compares the score of the test-taker to those in the NIH Toolbox nationally representative normative sample at the same age, where a score of 100 indicates performance that was at the national average for the test-taker participant’s age. Age-corrected standard scores were derived separately for children (ages 3-17) and adults (ages 18-85). A score of 115 or 85, for example, would indicate that the participant’s performance is 1 SD above or below the national average, respectively, when compared with like-aged participants. Higher scores indicate better performance.

The CSS is an item response theory (IRT)-based score metric that is useful for monitoring longitudinal change. The CSS is derived from the examinee’s raw score on the test and does not rely on comparison to the examinee’s age-based reference group. On the NIH Toolbox cognition measures, a CSS of 500 is indexed to the median ability of 10-year-olds in the norming sample. 

The percentile score is the transformation of a participant’s age-adjusted standard score into percentile data. A percentile represents the percentage of people nationally above whom the participant’s score ranks based on age-adjusted standard score.

Statistical analysis

Data were compiled and analyzed using the statistical software Stata 18.0 (StataCorp LLC, College Station, USA). The data were first checked for normality of distribution using the Kolmogorov-Smirnov test. Demographic data were presented as mean and median. Thereafter, tests for statistical significance of the quantitative variables between the two groups were carried out using the student’s t-test for normally distributed parameters and the Wilcoxon signed-rank test for non-parametric parameters. The significance level was set at a p-value <0.05, with a confidence interval of 95%, β=0.2, and power considered at 80%.

## Results

Subjects characteristics

The demographic details of the cases and controls are given in Table 1. The mean age of the cases and controls was 19.37 ± 0.92 and 19.65 ± 0.99 years, respectively. The average post-COVID duration at the testing time was 1050 ± 227 days. Interestingly, 10 case subjects, as opposed to five control subjects, self-reported experiencing at least one cognitive symptom included in the survey, like fatigue, forgetfulness, loss of attention, depression, or sleep-related problems (Table 1).

**Table 1:** Demographic details of case and control subjects PCOS: Polycystic ovary syndrome

	Cases	Controls
Number of subjects	29 (15 male and 14 female)	29 (15 male and 14 female)
Age	19.37 ± 0.92	19.65 ± 0.99
Socioeconomic status	Medium	Medium
Educational status	Undergraduate	Undergraduate
Smoking history	Nil	Nil
Post-COVID duration at the time of testing (in days)	1050 ± 227	---
Duration of symptomatic COVID-19 in days (onset of symptoms till recovery)	8.62 ± 6.03	---
Vaccination status at the time of COVID-19 infection (in %)		
Fully vaccinated (2 doses)	17.2	---
One dose received	10.3	---
Unvaccinated	72.4	---
Current vaccination status (in %)		
Fully vaccinated (2 doses)	93.1	93.1
One dose received	6.9	3.4
Unvaccinated	0	3.4
Coexisting disease (in %)		
Hypertension	0	0
Diabetes	0	0
Chronic heart disease	0	0
Chronic respiratory disease	0	0
Hypothyroidism	3.4	0
PCOS	3.4	0
None	93.1	100
Treatment received during COVID-19 symptoms (in %)		
Paracetamol	93.1	---
Azithromycin	51.7	---
Hydroxychloroquine	17.2	---
Ivermectin	20.7	---
Corticosteroids	6.9	---
Lopinavir/Ritonavir	0	---
Interferon B	0	---
Tocilizumab	0	---
Favripiravir	3.4	---
Vitamins	3.4	---
Symptoms at the time of cognitive testing (%)		
Fatigue	17.2	3.4
Forgetfulness	20.6	3.4
Loss of attention	20.6	13.8
Depression	6.9	0
Sleep-related problems	13.8	13.8
Other	0	0

Cognitive functions 

The cognitive performances were compared between cases and controls. Age-adjusted standard scores between cases and controls did not differ in either of DCCS (p=0.94), FICA (p=0.09), LSWM (p=0.31), PCPS (p=0.10), or PSM (p=0.70) tests. Age-adjusted fluid composite cognitive score, which signifies the fluidic ability of the person to solve problems in novel situations, also did not differ between cases and controls (p=0.19) (Table 2). 

**Table 2:** Comparison of age-adjusted standard scores between cases and controls Values are given in mean ± SD. ^a ^Cohen’s d: calculated as the difference in means divided by the common SD. ^b ^P-values: from two-sample t-tests. DCCS: Dimensional Card Change Sort; FICA: Flanker Inhibitory Control and Attention; LSWM: List-Sorting Working Memory; PCPS: Pattern Comparison Processing Speed; PSM: Picture Sequence Memory; FCS: Fluid Composite Score

Test Name	Cases (n=29)	Controls (n=29)	t-statistic	Effect Size ^a^	P-value ^b^
DCCS	102.1 ± 12.6	102.3 ± 11.0	0.08	0.02	0.94
FICA	111.9 ± 13.8	106.4 ± 10.8	1.70	0.44	0.09
LSWM	105.5 ± 12.2	100.8 ± 21.4	1.03	0.27	0.31
PCPS	106.4 ± 11.9	100.7 ± 14.1	1.66	0.43	0.10
PSM form A	108.1 ± 11.1	107.1 ± 8.4	0.39	0.10	0.70
FCS	110.8 ± 11.3	106.8 ±11.8	1.32	0.34	0.19

CSS, which is independent of the examinee’s age-based reference group, also did not differ between cases and controls in either DCCS (p=0.89), FICA (p=0.14), LSWM (p=0.73), PCPS (p=0.11), PSM (p=0.73), or FCS (0.22) (Table 3). 

**Table 3:** Comparison of change sensitive scores between cases and controls Values are given in mean ± SD. ^a^ Cohen’s d: calculated as the difference in means divided by the common SD. ^b^ P-values: from two-sample t-tests. DCCS: Dimensional Card Change Sort; FICA: Flanker Inhibitory Control and Attention; LSWM: List-Sorting Working Memory; PCPS: Pattern Comparison Processing Speed; PSM: Picture Sequence Memory; FCS: Fluid Composite Score

Test Name	Cases (n=29)	Controls (n=29)	t-statistic	Effect Size ^a^	P-value ^b^
DCCS	544.4 ± 29.2	545.3 ± 26.7	0.13	0.03	0.89
FICA	536.8 ± 18.3	530.4 ±14.4	1.48	0.38	0.14
LSWM	525.9 ± 15.5	524.5 ± 16.2	0.35	0.09	0.73
PCPS	560.7 ± 24.6	549.2 ± 29.9	1.60	0.42	0.11
PSM form A	525.9 ± 17.4	524.5 ± 13.2	0.35	0.91	0.73
FCS	538.9 ± 12.5	534.8 ± 13.1	1.24	0.32	0.22

There was also no statistically significant difference in the percentile scores. The mean ± SD of national age-adjusted percentile scores of FCS, which indicates global cognitive functioning, were 72.7 ± 20.1 and 63.7 ± 23.5 for cases and controls, respectively. However, no statistical significance was found in the FCS (p=0.11) percentile score between cases and controls (Table 4).

**Table 4:** Comparison of national age-adjusted percentile scores between cases and controls Values are given in mean ± SD. ^a^ Cohen’s d: calculated as the difference in means divided by the common SD. ^b^ P-values: from two-sample t-tests. DCCS: Dimensional Card Change Sort; FICA: Flanker Inhibitory Control and Attention; LSWM: List-Sorting Working Memory; PCPS: Pattern Comparison Processing Speed; PSM: Picture Sequence Memory; FCS: Fluid Composite Score

Test Name	Cases (n=29)	Controls (n=29)	t-statistic	Effect Size ^a^	P-value ^b^
DCCS	52.9 ± 26	53.5 ± 22.3	0.09	0.02	0.92
FICA	71.8 ± 22.5	63.6 ± 22.8	1.38	0.36	0.17
LSWM	61.8 ± 24.9	58.9 ± 26.5	0.42	0.11	0.66
PCPS	65.1 ± 22.5	51.5 ± 30.6	1.92	0.51	0.05
PSM form A	67.2 ± 23.3	65.8 ± 18.3	0.26	0.07	0.79
FCS	72.7 ± 20.1	63.7 ± 23.5	1.58	0.41	0.11

In conclusion, no statistically significant differences in cognitive function were observed between cases and controls in the study.

## Discussion

This study investigated the relation between cognitive functions (cognitive flexibility, attention span, inhibitory control, episodic memory, working memory, and processing speed) and COVID-19 infection in the long term (1050.689 ± 227.180 days from the date of diagnosis). 

The results of cognitive function testing indicated no significant cognitive impairment in cases as compared to age- and sex-matched controls, indicating that no significant association exists between mild COVID-19 infection and decline of cognitive functions in the long term. However, subjective symptoms of fatigue, forgetfulness, loss of attention, and depression were different between cases and controls. A study by Ryan et al. demonstrated similar findings to our study. Using the NIH Toolbox battery of tests, they found no difference in cognitive function in post-COVID-19 patients compared to controls seven months post-infection. However, they did find poorer emotional health and motor functions among post-COVID patients than controls [12]. 

In our earlier study, we showed a selective impairment of auditory working memory without any impairment of attention of visual working memory in medical students who suffered COVID-19 infection at least six months prior to the study [13]. Likely, this observation was related to the short-term post-infection in which the study was carried out, as opposed to the current study, which was carried out after a period of more than two years after infection. Few other studies have also reported mild impairment of short-term memory and attention in a comparatively younger population, even after four months of recovery from COVID-19 infection [14,15].

The pathogenesis of acute COVID-19 invasion progresses with the binding of spike (S) glycoprotein to the angiotensin-converting enzyme (ACE2) receptor present primarily on lung epithelial cells, leading to the blockade of innate immunity and evasion of adaptive immunity, followed by subsequent acute and long-term sequelae [2]. In relatively mild COVID-19 infections, there is a potent antiviral response, which results in decreased severity of symptoms and swift clinical resolution [16]. In contrast, in severe COVID-19 infections, unbridled SARS-CoV-2 replication results in aberrant proinflammatory responses and immune cell invasion [17], characterized by increased production of potent inflammatory cytokines such as tumor necrosis factor (TNF) and interleukin-6 (IL-6) [18]. There is considerable evidence that long-term tissue damage and persistent pathological inflammation associated with increased levels of circulating inflammatory cytokines result in tissue damage and associated long-term sequelae of COVID-19 infection [4,19,20]. Similarly, various studies have found associations between neuroinflammation, aberrant cell-mediated immune response, neuroinvasion by virus cells, as well as microthrombotic complications, and persistent neurological sequelae in recovered COVID-19 patients [19-21]. SARS-CoV-2 has hence been described as a neuroinvasive, neurotropic, and neurovirulent virus [19], with the ability to cause neurological manifestations by both direct as well as indirect mechanisms. The direct mechanisms involve invasion of the central nervous system (CNS) by the virus, and the indirect mechanisms include associated inflammation and dysregulated immune response as well as microglial cell dysregulation. Interestingly, certain anatomically vulnerable regions of the brainstem have been demonstrated to possess expression of ACE2 receptors [22], which may indicate a possible site for invasion of the CNS by the virus, and have been implicated in cases of respiratory failure associated with COVID-19 infection [23]. Another study demonstrated possible disruption to micro-structural and functional brain integrity in the recovery stages of COVID-19, suggesting a plausible mechanism for the long-term consequences of SARS-CoV-2 [24]. In short, the mechanism behind long-term neurological sequelae in SARS-CoV-2 infection consists of a complex interplay of inflammatory, immune-mediated, and vascular damage. 

In a study that correlated COVID-19 disease severity with inflammatory markers and multi-omic profile in the body [25], a sharp shift in disease state from mild to moderate was associated with preferential loss of lipid, amino acid, and xenobiotic metabolism, as well as significant elevation of inflammatory cytokines. Similarly, an increase was observed in unusual clonally expanded phenotypes of peripheral immune cells. Another study found that inflammatory markers such as IL-6, C-reactive protein (CRP), ferritin, lactate dehydrogenase (LDH), and D-dimer were found to be highly elevated in severe and critical COVID-19 infection as opposed to mild infection [26]. These findings indicate that mild infection with SARS-CoV-2 presents with a relatively forgiving inflammatory response as well as lower levels of immune activation, which could be of significance in decreased persistent neuroinflammation and immune response in the long run, hence reducing the chances of neurological sequelae.

A particular aspect of the young adult brain that bears mention in the context of brain injury is that of neural plasticity. Neural plasticity refers to the capacity of the nervous system to modify itself, functionally and structurally, in response to experience and injury [27]. Physiological changes in response to brain injury that occur as a part of this phenomenon involve mechanisms such as long-term potentiation and depression, as well as alterations of levels of excitatory and inhibitory neurotransmitters [28]. Associated anatomical changes are dendritic branching, axonal sprouting, and synaptogenesis. All these changes occur at rapid rates in the event of brain injuries, facilitating recovery of function of affected areas of the CNS. Interestingly, a study utilizing transcranial direct current stimulation to determine age-related differences in motor cortex plasticity in adults concluded that a decline of long-term potentiation (LTP)-like plasticity at higher age could contribute to cognitive deficits observed in aging [29]. As a result of this higher capacity for neuronal plasticity in younger age, recovery from the subacute neurological sequelae observed in the short term [30] might occur within months to years following recovery from the acute phase of mild COVID-19 infection. This, in turn, explains the lack of correspondence between the results of cognitive functions in the short and long term.

Taking everything into account, the positive results obtained with respect to the lack of long-term neurological sequelae in young adult COVID-19 patients with mild infection can be explained by a combination of suppressed aberrant immune response of the body as well as the remarkable adaptive capability of the young adult brain. Although it cannot be said with certainty that the young human brain is not susceptible to damage by infection with SARS-CoV-2 except in high levels of disease severity, the absence of persistent neurological deficits in these individuals certainly presents an optimistic outlook to understanding the processes behind the occurrence of these problems in other groups of individuals, as well as in developing appropriate therapeutic interventions for the same. 

Limitations

The results of this study cannot be exactly generalized to all scenarios. First of all, the study was carried out with a relatively small sample size of 58, and a convenient sampling technique was used to include the study subjects. Further investigations involving a larger population and dataset would be required to optimally describe the effects seen in the population at large. Secondly, the population under study, undergraduate medical students from a large tertiary care center, represents a specific group of individuals whose cognitive capabilities may differ from those of the general population in the absence of any offending factor. This may have resulted in a sampling bias, and the results obtained with these individuals may not correlate with that of results obtained from another group of individuals. Furthermore, the results of the demographic data may not be completely accurate, as the complaints of current problems in cognitive function were self-reported by participants.

Additionally, recall and self-reporting bias may be present, given that the collection of history and demographic data was based on a survey rather than a comprehensive analysis of the medical records of the participants. There may also be misclassification bias regarding mild COVID-19 infection, primarily due to the lack of patients with less severe symptoms who actually sought medical attention during the pandemic, leading to a scarcity of medical records to confirm the existence of a mild infection.

## Conclusions

The results of this study robustly demonstrate that cognitive impairment was not evident in young adults who had previously symptomatic mild COVID-19 infection at least two years prior. Therefore, it can be concluded that cognitive performance in all domains of cognitive function, such as executive function, cognitive flexibility, attention, episodic memory, and processing speed, did not differ between healthy controls and young adults with prior mild COVID-19 infection. A decline in cognitive functions appears to be linked to the clinical severity of the disease as well as the overall health and immune status of the patient. In the younger population, the effects of mild COVID-19 on cognition may be self-resolving.

Considering cognitive functions a key determinant of quality of life, especially in the younger population, this study indicates a good clinical outcome in younger patients with mild COVID-19 infection in the long term. However, it must be noted that the absence of findings in this study cohort does not rule out potential cognitive impairment in other populations (e.g., older adults or those with more severe COVID-19). Long-term studies with larger and more diverse populations are needed to definitively conclude that mild COVID-19 infection does not have lingering cognitive effects in younger adults.
